# Activation Kinetics and Off-Target Effects of Thymus-Initiated Cre Transgenes

**DOI:** 10.1371/journal.pone.0046590

**Published:** 2012-10-01

**Authors:** Jianjun Shi, Howard T. Petrie

**Affiliations:** The Scripps Research Institute, Jupiter, Florida, United States of America; Baylor College of Medicine, United States of America

## Abstract

The bacteriophage enzyme Cre is a site-specific recombinase widely used to delete loxP-flanked DNA sequences in lineage-specific fashion. Several mouse lines that direct Cre expression to lymphoid progenitors in the thymus have been established, but a side-by-side comparison of when they first become active, and/or their relative efficiency at various developmental stages, has been lacking. In this study, we evaluated these in four common Cre transgenic strains with thymus-initiated promoters (Lck, Cd2, or Cd4). We found that while all of them eventually labeled nearly all thymocytes, their kinetics were dramatically different, and other than Cd4[Cre], did not faithfully recapitulate the expression pattern of the corresponding endogenous gene. Perhaps even more importantly, while thymuses from some strains compared favorably to thymuses from control (Cre-negative) mice, we found that Cre expression could also result in off-target effects, including moderate to severe decreases in thymic cellularity. These effects occurred in the absence of loxP-flanked DNA target genes, and were dose and copy number dependent. Loss of cellularity was attributable to a specific decrease in CD4^+^8^+^ immature cells, and corresponds to an increased rate of programmed cell death. In addition to a comprehensive analysis of activation kinetics in thymus-initiated Cre transgenes, our data show that Cre is toxic to CD4^+^8^+^ cells in a dose-dependent fashion, and emphasize that the choice of thymus-initiated Cre strain is critically important for minimizing off-target effects of Cre.

## Introduction

The thymus is the primary site of lifelong T lymphopoiesis. The thymus contains no self-renewing progenitor cells, but instead relies on the semi-continuous recruitment of multipotent progenitors that can be found circulating in the bloodstream. Once these cells enter the thymus, microenvironmental conditions unique to that organ coordinate progression through a complex series of developmental transitions, including progressive loss of non-T lineage potential, acquisition of multiple asymmetric T lineage fates and functions, and extensive proliferative expansion of each thymic homing cell, as reviewed in [1]. The ability to perform genetic perturbation of specific processes at specific stages has greatly facilitated an understanding of the mechanisms of these processes in all systems of differentiation. A substantial number of mouse lines have been generated where expression of the bacteriophage DNA recombinase Cre is directed to T lymphocytes via T lineage-specific promoters, several of which are first activated in the thymus. The underlying assumption is that transgenic Cre expression will mirror the transcriptional activity of the corresponding endogenous gene, although in many cases, the validity of that assumption has not been clearly established.

Expression of Cre recombinase is unquestionably the most widely used approach for conditional deletion of mammalian DNA sequences, after flanking them with the corresponding 34 bp Cre recognition site (denoted loxP). Because the loxP sequence site does exist with measurable frequency in mammalian genomes, it is generally assumed that mammalian expression of Cre, in the absence of engineered loxP sites, is essentially irrelevant. However, numerous studies have indicated that Cre may have off-target effects, for examples, see [2], [3]–[6], including one indication that Cre may have unintended effects in thymocytes [7]. Obviously, the potential for off-target effects of Cre, in thymocytes or in cells of any lineage, warrants careful evaluation of the transgenic models available, and of the timing and magnitude of these effects when Cre is driven by various lineage-specific reporters.

In the present study, we conduct a side-by-side comparison of the kinetics of lymphoid-restricted Cre activity in the most commonly used thymus-initiated transgenic strains. We find that activation in most of them, as assessed by crossing to two different reporter strains harboring a conditional fluorescent protein gene, differs substantially from the transcriptional activity of the corresponding endogenous gene. Further, we find that Cre itself exhibits off-target effects that can result in a very substantial thymic phenotype, manifest as a reduction in cellularity that corresponds to a decrease in CD4/CD8-double positive (DP) cells, and an increase in programmed cell death. These phenotypes are not universal to all thymus-initiated lymphoid Cre transgenes, at least in hemizygous form, but are clearly dose-dependent, with the most prominent effects occurring in the strains with higher levels of Cre. Together, our results indicate that selection of the appropriate Cre transgene is essential for both the correct timing of conditional deletion, and for minimizing the impact of very substantial off target effects of Cre in thymic lymphoid cells.

## Materials and Methods

### Mice

This study was carried out in strict accordance with the recommendations in the Guide for the Care and Use of Laboratory Animals of the National Institutes of Health. The relevant animal protocol was approved by the Animal Care and Use Committee of the Scripps Research Institute. Experimental mice were bred at Scripps-Florida, were of mixed sexes, and were used at 5±1 weeks of age. C57BL6 breeders were purchased from Taconic. Breeders for the Lck[Cre] transgenic strain originating from the laboratory of Dr. Jamey Marth [8] were purchased from Jackson Laboratory. Breeders for the Lck[Cre] transgenic strain originating from the laboratory of Dr. Chris Wilson [9] were purchased from Taconic. Cd2[Cre] [10] and Cd4[Cre] [9] transgenic breeders, as well as breeders for the ROSA[(stop/flox)YFP] reporter strain [11], were purchased from Jackson Laboratory. Breeders for the ROSA[(stop/flox)tdRFP] reporter strain were the kind gift of Dr Hans Joerg Fehling [12].

### Single cell suspensions, cell counting, and flow cytometric analysis

Upon euthanasia, thymuses were removed and placed immediately in ice-cold medium (Hank's balanced salt solution, 5% FBS, 10 µg/ml DNAse, 0.01% sodium azide). All subsequent steps were performed at 4°C. Thymic lobes were lacerated with a scalpel and pressed through a wire mesh using the plunger from a 3 ml syringe. Cells suspensions were counted using a hemacytometer; viability was determined by eosin exclusion. Cell suspensions were stained with a cocktail of antibodies recognizing the appropriate surface markers as previously described [13], [14]. The TUNEL assay was performed as previously described [15]. Briefly, stained cells were washed once in PBS and then resuspended in a solution of 2% CH_2_O/PBS for 2 hrs on ice. Fixed cells were washed once in PBS and resuspended in PBS containing 5% FBS and 0.5% Tween-20 for 30 min on ice, followed by 2 washes in PBS. Fixed, permeabilized cells were then subjected to end-labeling of DNA strand breaks using FITC-dUTP and terminal deoxynucleotidyl transferase, exactly as described by the manufacturer in the BD Pharmingen Apo-Direct kit (cat. #556381), except that 4′6-diamidino-2-phenylindole (DAPI, 10 µg/ml) was used for DNA counterstaining instead of propidium iodide. For intracellular detection of Cre protein, fixed, permeabilized cells as above were stained with a biotin-labeled anti-Cre antibody (Covance BIOT-106L), followed by secondary detection using a streptavidin fluorochrome. Stained cells were analyzed on an LSR2 analytical cytometer (BD Immunocytometry Systems) equipped with 350 nm, 405 nm, 488 nm, 561 nm, and 633 nm lasers.

## Results

### In vivo kinetics of reporter activation induced by various thymus-initiated Cre transgenes

**Figure 1:**
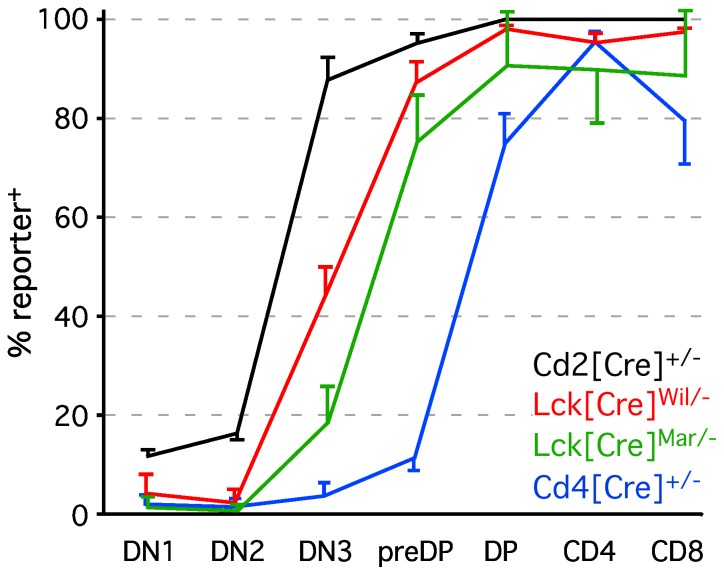
In vivo kinetics of reporter activity induced by various thymus-initiated Cre transgenes. The percent of reporter^+^ cells at all major stages of thymocyte differentiation are indicated. All results except those from Cd2[Cre] include pooled data from ROSA[(stop/flox)tdRFP] and ROSA[(stop/flox)YFP] reporters, which were essentially indistinguishable from each other in all three Cre strains tested; due to the absence of any reporter strain variation, Cd2[Cre] was only tested using the ROSA[(stop/flox)tdRFP] reporter. Each data point represents the mean ± s.d. for 8–14 individual experiments (thymuses) performed over a 3-year period. All mice were hemizygous for the Cre transgene, and heterozygous for the reporter allele. Note that with the exception of Cd4, none of these Cre transgenes faithfully reflects temporal expression of the corresponding endogenous gene (see Figure S1).

Various thymus-initiated Cre transgenes have been generated, including Cd2[Cre] [10], Cd4[Cre] [9], and two different strains of Lck[Cre], one generated by the Wilson laboratory, designated here as Lck[Cre]^wil^ [9], and one generated by the Marth laboratory [8], designated here as Lck[Cre]^mar^. However, their relative temporal and quantitative activities have not been evaluated. Further, it is generally assumed that all are expressed in a manner similar to that of their endogenous counterparts (e.g., Lck, for Lck[Cre], etc.), although with few exceptions, this has not been tested directly, and is an important criterion in the selection of a Cre transgenic model. We therefore crossed mice carrying various thymus-initiated Cre transgenes with two different mouse strains carrying conditionally activated fluorescent reporter alleles [11], [12]. The results of these studies are shown in Fig. 1. All Cre strains were essentially reporter negative at the earliest CD4/CD8 double negative stage (DN1, lin^−^CD25^−^CD117^+^) except for Cd2[Cre], which marked about 10% of DN1 cells, suggesting that Cd2[Cre] may either become active before homing to the thymus, or may be expressed at very low levels in DN1. Our gene expression analysis (Fig. S1) suggests that Cd2 gene expression is virtually undetectable before the DN3 stage (lin^−^CD25^+^CD117^−^), and since Cd2[Cre] activity also marks early B lymphoid progenitors [10], we conclude that the former is more likely to be the case. This is further supported by the finding that reporter^+^ cells are found at very similar frequencies in both DN1 and DN2 cells (lin^−^CD25^+^CD117^+^), and do not increase in proportion until the DN3 stage, where the endogenous Cd2 mRNA is also dramatically upregulated (Fig. S1). We note that the proportion of DN1 and DN2 cells marked by Cd2[Cre] activity differs between our study and that shown in the original description [10], a difference we attribute to the fact that those authors used CD44, rather than CD117, to identify DN1 and DN2, thus including many cells of indeterminate lineage [13]. Our data from DN3 onwards are in complete agreement with the prior study, indicating around 90% labeling efficiency in DN3 cells, and virtually all cells labeled by the preDP stage (CD4^lo^CD8^lo^CD25^−^CD117^−^).

In contrast to Cd2[Cre], other thymus-initiated Cre transgenes showed almost no detectable labeling at the DN1 or DN2 stage. For Cd4[Cre] this is to be expected, since Cd4 transcription begins very early in the CD4/CD8 double positive stage (preDP, CD25^−^117^−^lin^low^), see Fig. S1, and [16]. However, endogenous Lck is expressed at very high levels even in the earliest DN1 cells (Fig. S1), consistent with findings from a previously published results of RT-PCR or Northern blotting [17], [18]. Nonetheless, Cre expressed under the Lck proximal promoter, revealed minimal reporter activation at the DN1 and DN2 stages in both Lck[Cre]^wil^ and Lck[Cre]^mar^ strains. This is consistent with lack of activity in other previously published studies using the Lck[Cre]^wil^ strain [19], [20], both of which also showed levels of induction similar to ours at the DN3 stage (approximately 50% of cells labeled, Fig. 1), followed by nearly complete labeling by the preDP stage. In contrast, the Lck[Cre]^mar^ strain exhibited only ∼20% labeling at the DN3 stage, and about 75% at the preDP stage. Since both of these transgenes used nearly identical promoter constructs (proximal Lck promoter), we attribute the enhanced efficiency of the former to eukaryotic optimization of the translation start site and nuclear localization signal in Cre [9]. Overall, while all promoters studied resulted in nearly complete labeling by the DP stage, each had a distinct kinetics of labeling, and none, other than Cd4, faithfully recapitulated expression of the corresponding endogenous mRNA.

### Expression of most thymus-initiated Cre transgenes leads to reduced thymic cellularity

**Figure 2:**
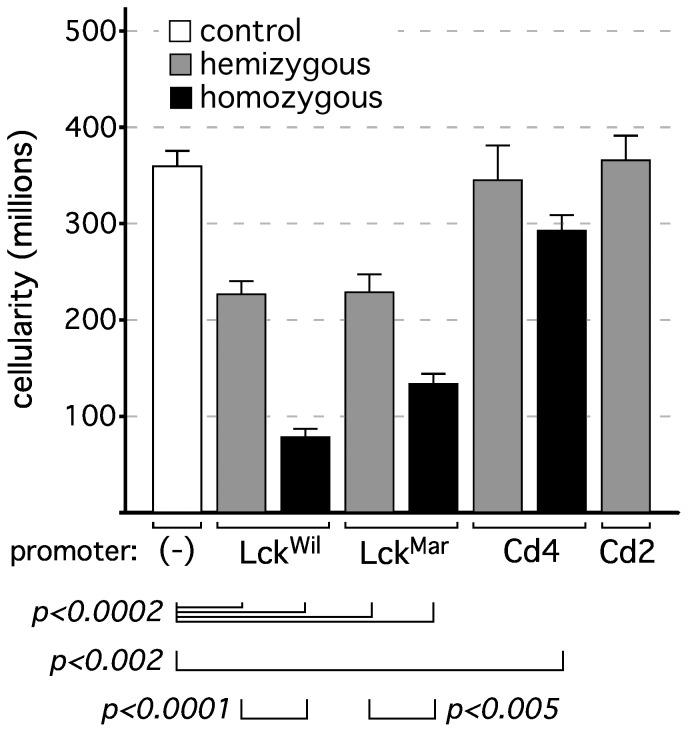
Reduced thymic cellularity in thymus-initiated Cre-transgenic strains. Each bar represents the mean ± s.d. for 6–13 individual experiments (thymuses); mice were males and females, 5 weeks of age. Statistics represent the Student's two-tailed t test for unpaired samples. In mice hemizygous for the a Cre allele, both Lck strains exhibited substantial reductions in thymic cellularity, while Cd2[Cre] and Cd4[Cre] did not. Mice that were homozygous for Cre alleles revealed a further exacerbation of this phenotype that was significant even in previously unaffected Cd4[Cre] mice (note: Cd2[Cre] homozygous mice were not generated, due to embryonic lethality). Since no loxP target sequences were present, these data suggest the existence of off-target effects of Cre in developing thymocytes.

In the course of using Cre transgenes for thymus-specific reporter activation, we noted empirically that thymuses from Cre-only mice (no floxed target) were frequently smaller than age-matched wildtype or loxP-only controls. To further evaluate this, we measured thymic cellularity in mice carrying various thymus-initiated Cre transgenes. Initially, we only evaluated hemizygous mice, and found that while hemizygous Cd2[Cre] and Cd4[Cre] transgenes resulted in cellularity indistinguishable from that of wildtype controls, both Lck[Cre] strains tested resulted in about a 40% loss of cellularity, even in the hemizygous state (Fig. 2). The reason for the differences between Cre strains was unclear, but we speculated that it might relate to different levels of Cre in the different transgenes. Since the relative levels of Cre protein were not known, we first evaluated the consequence of doubling Cre expression by using homozygous mice (note: as per the donating investigator, Cd2[Cre] is lethal, for unexplained reasons, in homozygous mice, see the Health & Husbandry tab on the Jackson Laboratory webpage for strain 008520). In all cases tested, homozygosity for Cre resulted in a further reduction in cellularity; in the case of Lck[Cre]^wil^, cellularity was reduced to about 25% of control levels, and Lck[Cre]^mar^ to about 35%. Even Cd4[Cre], which displayed no phenotype in the hemizygous state, showed a significant reduction of about 20% in the homozygous state. Together, these data suggest that Cre itself may lead to off-target phenotypic effects in a dose dependent fashion, a conclusion that is further strengthened in the next section.

### Reduced thymic cellularity in the presence of thymus-initiated Cre transgenes correlates with Cre protein level

**Figure 3:**
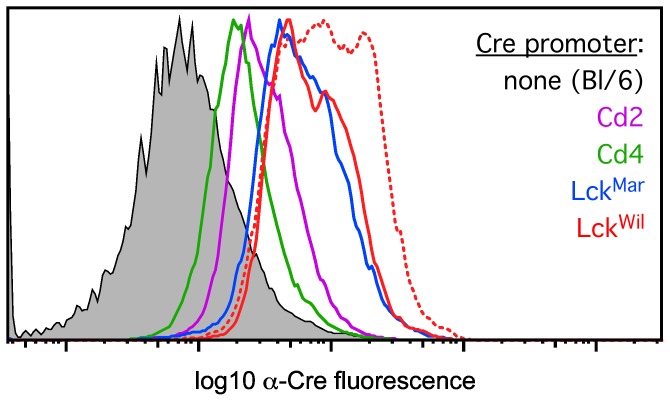
Intracellular Cre protein levels correlate with reduced thymic cellularity in thymus-initiated Cre-transgenic strains. Thymocytes from control (C57Bl/6) mice, or from mice expressing various Cre-transgenes, were isolated, fixed, and stained with an antibody recognizing the Cre protein, followed by flow cytometric analysis. Levels of intracellular Cre correlated directly with the appearance of phenotypic effects, with lowest levels in the Cd4[Cre] and Cd2[Cre] strains, and highest levels in both Lck[Cre] strains. Homozygous mice for the highest expressing lck[Cre] strain (Lck[Cre]^Wil^, dashed line) predictably expressed even higher levels of Cre than hemizygous mice, and had the most profound phenotype (Figs. 2 and 4). These results suggest that above a certain threshold, Cre protein is toxic to thymocytes in a dose-dependent manner. These experiments were repeated at least four times with essentially identical results.

To determine whether the cellularity phenotype seen in response to certain Cre transgenes and doses correlated with absolute levels of Cre, we measured intracellular Cre protein by flow cytometry, using a Cre-specific antibody. Since virtually all strains tested labeled almost all cells by the DP stage, we used unfractionated thymocytes, which are >95% DP (or later) cells. As is shown in Fig. 3, loss of cellularity (Fig. 2) was directly correlated with Cre protein levels in hemizygous mice, as we found lower Cre protein staining levels in Cd2[Cre] and Cd4[Cre], higher levels in Lck[Cre]^mar^, and the highest levels in Lck[Cre]^wil^. Notably, thymocytes from homozygous Lck[Cre]^wil^ mice had even higher levels of Cre (dashed line; other homozygous mice are not shown, for clarity), supporting the notion that homozygosity increases Cre protein levels, and that Cre protein levels correlate with phenotypic effects on thymic cellularity, since this was also the most severe off-target phenotype (Fig. 2).

### Reduced thymic cellularity in response to Cre transgenes correlates with a specific loss of DP thymocytes

**Figure 4:**
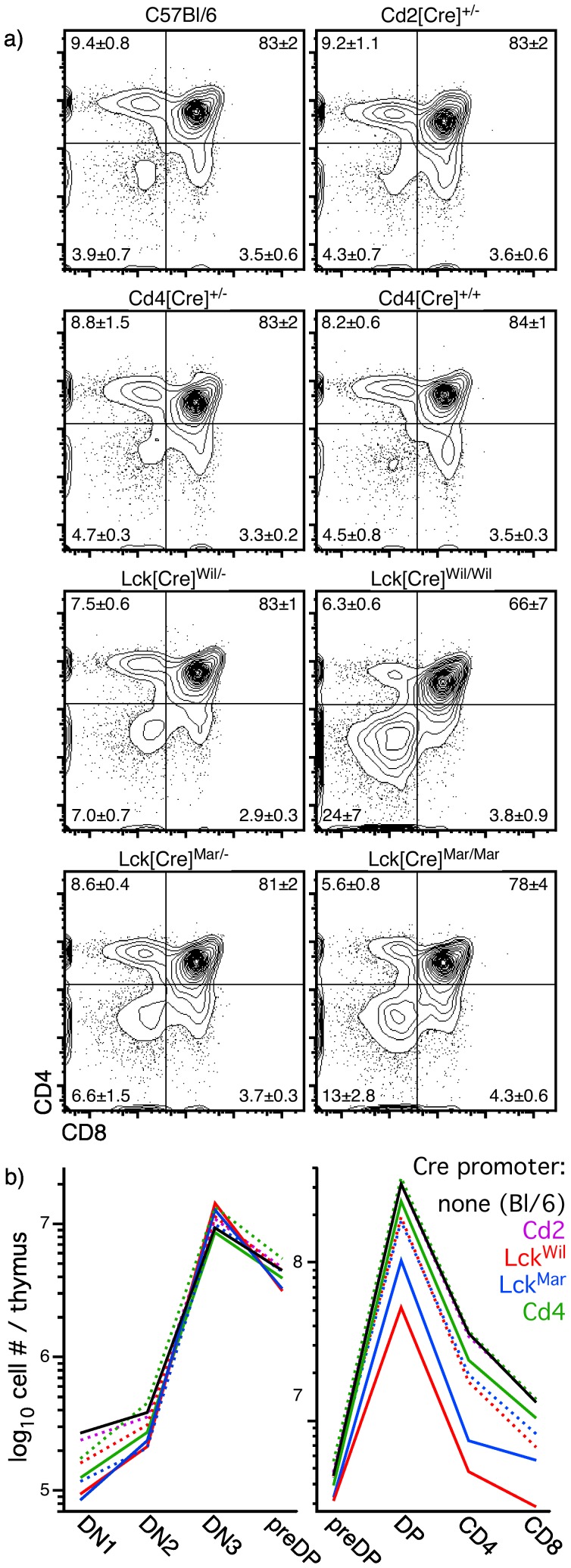
Thymus-initiated Cre expression results in a loss of DP cells. Panel a) shows the relative proportions of various populations identified by CD4 and CD8 staining on thymocytes from various types of Cre transgenic mice, or C57Bl/6 controls. Statistics indicate mean ± s.d. for 3–9 independent experiments. Panel b) shows absolute numbers of cells per thymus for each major stage of differentiation in the same mice (dashed line  =  hemizygous; solid line  =  homozygous); note that because of extensive overlap, only the mean value is shown. Absolute numbers of early progenitor stages were very similar in all strains of mice, suggesting that the effects of Cre were minimal among at these stages. In contrast, substantial differences were seen at the transition to the DP stage. Both strains of hemizygous mice expressing Cre under the Lck promoter showed substantial reductions in DP cell number, changes that are exacerbated in homozygous mice of these strains. These data are in complete agreement with cellularity data shown in Fig. 2, and suggest that the presence of Cre and, in particular, the absolute levels of Cre, may be toxic to DP thymocytes.

In order to further characterize the loss of thymic cellularity seen in some Cre transgenic strains, we evaluated the proportions of cells at classical stages of thymocyte development, as defined by CD4 and/or CD8 surface expression. Consistent with the absence of a change in cellularity, no defects in CD4/CD8 distribution were seen in hemizygous Cd2- or Cd4-driven Cre (Fig. 4), and despite a small reduction in cellularity in homozygous Cd4[Cre] mice (Fig. 2), no detectable changes in CD4/CD8 distribution were seen. However, both Lck[Cre] strains exhibited observable changes in phenotype, most easily recognized in hemizygous mice as an increase in DN cells, and readily seen in homozygotes as a decrease in DP cells. Since a decrease in DP cells can result in an apparent increase in DN cells, and in order to further determine the staging of Cre-mediated off-target effects, we measured absolute numbers of thymocytes at all stages of development in the various Cre strains. As can be seen in Fig. 4b, the numbers of early progenitor cells (DN1, DN2, DN3, preDP) were nearly identical in all strains of mice or controls, regardless of whether the transgene was hemizygous or homozygous. However, at the DP stage, all Cre strains were statistically different from controls (p<0.02, Student's two-tailed t test for independent samples) except for hemizygous Cd2[Cre] or Cd4[Cre] strains, in complete concordance with cellularity data (Fig. 2). In all cases, the loss of DP cellularity seen in homozygous mice was more severe than that seen in hemizygous mice. Mature single positive (SP) cells were found in constant proportion to DP cells in all strains of mice. Together, these data suggest that DP cells bear the brunt of the off-target effects of Cre, and are primarily responsible for the observed changes in cellularity and CD4/CD8 distribution.

### Thymic expression of Cre protein results in an increased propensity towards cell death

**Figure 5:**
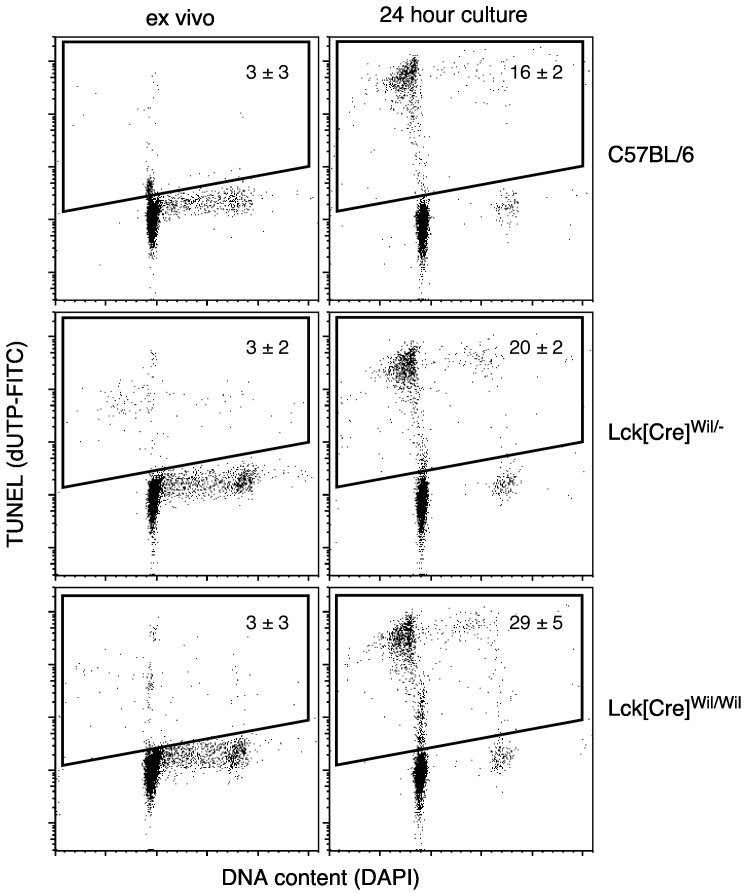
Phenotypes resulting from Cre expression in thymocytes correlate with an increased propensity towards cell death. To test whether Cre protein is toxic to thymocytes in a dose-dependent fashion, analysis of DNA double strand breaks (TUNEL) was performed on wildtype thymocytes or thymocytes from mice carrying one (hemizygous) or two (homozygous) Lck[Cre] transgenes. Freshly isolated thymocytes were indistinguishable from the three types of mice, suggesting that clearance of apoptotic cells from the thymus was not affected. However, when thymocytes were cultured overnight in medium, the presence of Cre resulted in an increase in cell death that was proportional to the amount of Cre protein expressed. These results further strengthen the conclusion that Cre protein is toxic to thymocytes in a dose-dependent manner. Data represent mean ± s.d. for 3–4 independent experiments for each mouse type.

The loss of cellularity phenotype (Fig. 2) could result either from a block in differentiation, decreased proliferation, or increased cell death. A block in differentiation by Cre seemed unlikely, and since DP cells proliferate little but are highly susceptible to cell death, we tested the hypothesis that reduced cellularity in affected strains occurred as a result of increased cell death. As can be seen in Fig. 5, very little difference was seen ex vivo in control mice vs. mice hemizygous or homozygous for the most affected strain (Lck[Cre]^wil^), consistent with the fact that apoptotic cells are rapidly cleared from the thymus in vivo. Upon short-term in vitro culture, however, clear differences were seen, with approximately a 25% increase in TUNEL^+^ cells at 24 hours in hemizygous thymocytes, and an almost twofold increase in homozygous thymocytes. These data further substantiate a dose-related effect of Cre, and indicate that loss of cellularity results from a Cre-induced increase in cell death.

## Discussion

In this manuscript, we present an extensive side-by-side analysis of the kinetics of reporter activation using several common thymus-initiated, lymphoid-targeted Cre transgenes. Several previously published studies have addressed the timing of several of these Cre transgenes singly, or at some of the developmental stages described here, for examples, see [9], [19]–[21], [10], but a comprehensive side-by-side comparison has been lacking. The assessments that have been published generally correlate well, both temporally and quantitatively, with our present findings (Fig. 1). The exception was the DN1 stage, which was highly variable. We note that most of these studies were performed before extensive heterogeneity within the DN1 subset became widely known, and used CD44, rather than CD117 (or, ideally, CD44 plus CD117), to define DN1 cells. It is now widely recognized that the previous definition of DN1 (CD25^−^44^+^lin^−^) includes a substantial proportion of atypical T lineage progenitors and/or non T-lineage cells, characterized by kinetics of differentiation, proliferative capacities, and lineage potentials [13] that differ from canonical T cell progenitors. In contrast, CD25^−^44^+^117^hi^lin^−^ DN1 cells, as analyzed here, exclusively represent cells in the mainstream of T lineage differentiation in the thymus.

Our data indicate that Cd2[Cre] is the earliest activated thymus-initiated lymphoid transgene, with approximately 10% of cells labeled as early as the DN1 stage. We note that this level of labeling does not increase, as would be expected, at the DN2 stage, but instead remains nearly constant. We also note that Cd2[Cre] efficiently labels B lineage cells [10], indicating that pre-thymic activation of this transgene occurs. Since Cd2 mRNA is virtually undetectable at the DN1 and DN2 stages of differentiation (Fig. S1), and is barely detectable at the DN3 stage, we conclude that reporter labeling in DN1/DN2 cells is a consequence of pre-thymic activation of Cd2[Cre] that persists before further activation in the thymus, which occurs at the DN3 stage. In contrast, Lck transcription occurs at a very high level even at the DN1 stage, see Fig. S1 and [17], [22], yet reporter activation by Lck[Cre] is not evident until the DN3 stage Fig. 1 and [19], [20]. This finding is consistent in either strain of Lck[Cre] mice [8], [9], even though reporter activation at the DN3 stage differs by 2.5-fold between the two strains. Thus, although all of these strains efficiently label most thymocytes by the DP stage, none of them faithfully reflects the kinetics of expression of the endogenous gene. In the case of Cd2[Cre], this may be related to differences between the mouse Cd2 promoter and the human promoter used for the transgene construct [10], while in the case of Lck[Cre], it may be a consequence of the use of a minimal promoter [8], [9]. In this regard, the activity of Cd4[Cre], which used a transgenic construct including the endogenous murine Cd4 promoter, enhancer, and silencer [9], [23], does essentially parallel the expression of the endogenous mRNA (Fig. 1 and Fig. S1). Thus, our data indicate that the choice of Cre transgene is critical when intermediate stages of thymic lymphopoiesis are to be studied, and that other than Cd4[Cre], expression of the endogenous gene cannot be used to predict temporal activation of the transgene.

During our characterization of reporter activation in various thymus-initiated lymphoid Cre transgenes, we noted empirically that thymuses from some Cre transgenic strains were consistently smaller than others, even in age- and sex-matched animals. As shown in Fig. 2, these changes were significant and repeatable, but were not universal among Cre transgenic strains, at least in hemizygous mice. Note that none of the mice analyzed possessed engineered loxP target sequences, and thus, these were off-target effects of Cre. We considered two possible explanations for this, namely, that it was either a consequence of the timing of Cre induction (i.e., early vs. late), or was related to the absolute levels of Cre expression. Given that Cd2[Cre] is the first Cre transgene to exhibit activity in the thymus, but is the least affected by changes in cellularity (no significant change observed in hemizygotes), the former appears to be unlikely. Further, onset of expression would occur at the same time in both hemizygous and homozygous mice, which cannot explain the differences in phenotype between them (Fig. 2). In contrast, doubling the level of Cre, via generation of homozygous mice, resulted in a further exacerbation of the phenotype, even leading to a significant reduction in thymic cellularity in the Cd4[Cre] strain, where none was observed in hemizygous mice (note: Cd2[Cre] homozygous mice could not be generated; see Results). These data strongly suggested that Cre level, rather than the timing of Cre expression, was the cause of off-target effects. To evaluate this further, we used an antibody raised against Cre to quantitate relative levels of Cre by flow cytometry, and found that Cre levels correlated directly with the appearance of a loss-of-cellularity phenotype (Figs. 2, 3, 4). Reduction in thymic cellularity and the off-target effects of Cre appear to be focused on the DP stage (Fig. 4), which may be related to the intrinsic predisposition of these cells to succumb to programmed cell death.

Cre-mediated toxicity has been noted in multiple tissues and models, for examples, see [2], [3]–[6], including one passing report in the thymus [7], although the interpretation of that study, which used an ER:Cre fusion construct, is complicated by the fact that both lymphoid and stromal cells in the thymus express estrogen receptors, and respond to estrogen (or its analogs) by a decrease in cellularity, for one of many examples, see [24]. In virtually all studies reported, Cre-mediated toxicity was dose dependent, and led to an increase in cell death, identical to our findings in thymocytes. While the nature of this effect is not completely resolved, evidence suggests that it may result from the normal enzymatic activity of Cre at DNA sites with limited loxP sequence homology, and thus, would be expected to occur more frequently at higher Cre concentrations [2], [6], [25]. Such off-target activity can lead to genomic instability and chromosomal abnormalities [2], [6], and especially in cells such as thymic DP, which are poised to die, would be expected to lead to an increase in cell death, precisely explaining the phenotype that we observe in thymocytes.

Our data thus indicate that Cre can be toxic to thymocytes, primarily DP cells, leading to off-target effects and profound phenotypes even in the absence of loxP-targeted sequences. Similar to the caveat defined by each of the other studies cited above, we conclude that the only appropriate control for targeted deletion studies is the corresponding Cre-only transgene, and that studies that use(d) other types of controls (loxP only, wildtype) must be interpreted with suspicion in this light. Fortunately, we note that, at least in hemizygous form, both Cd2[Cre] and Cd4[Cre] appear to have minimal off-target effects, while remaining highly efficient at mediating deletion, albeit at very distinct stages of development. In contrast, both of the widely used Lck[Cre] proximal promoter strains exhibit a profound off-target effects, even in hemizygotes. Finally, we note that there does not appear to be an existing Cre transgenic model that is effective in targeting thymocytes very early during development (ideally, DN1), representing a rather substantial gap in the thymic toolbox. It would seem that a Cre knockin targeted to a gene expressed in DN1 cells, where promoter, enhancer, and other regulatory elements are preserved intact, would be the best approach, although our studies also suggest that selection of a gene expressed at slightly lower levels may be a better choice than a highly expressed gene.

## Supporting Information

Figure S1 **Temporal comparison of endogenous gene transcription vs. Cre transgene activity**. RNA was isolated from pooled, sorted C57BL/6 thymocyte populations, as shown, and used as template to probe MOE4302 gene arrays (Affymetrix, representing all known genes and ESTs). Gene expression data represent the mean MAS5 value for three independent gene chips at each stage, expressed as a percentage of maximum expression. Absolute maximum values were 18,142 for Cd2, 12,907 for Lck, and 3782 for Cd4, where the median (nominal present/absent cutoff) for all genes on the chip was set to 500. Where multiple probesets were present (n = 4 for Lck, n = 2 for Cd4), the corresponding probeset values were averaged. Reporter activity is derived from Figure 1, but is expressed as a percentage of maximum (without error bars) for comparison purposes. Only Cd4[Cre] activity appears to closely reflect the activity of its endogenous counterpart, while Cd2[Cre] activity is dramatically accelerated, and Lck[Cre] of either strain is substantially delayed.
(PDF)
