# Supplementary material for: Activation Kinetics and Off-Target Effects of Thymus-Initiated Cre Transgenes
Source: PLoS One. 2012 Oct 1;7(10):e46590. doi: 10.1371/journal.pone.0046590 (PMC3462198; doi:10.1371/journal.pone.0046590)
Supplement: Figure S1 — Temporal comparison of endogenous gene transcription vs. Cre transgene activity. RNA was isolated from pooled, sorted C57BL/6 thymocyte populations, as shown, and used as template to probe MOE4302 gene arrays (Affymetrix, representing all known genes and ESTs). Gene expression data represent the mean MAS5 value for three independent gene chips at each stage, expressed as a percentage of maximum expression. Absolute maximum values were 18,142 for Cd2, 12,907 for Lck, and 3782 for Cd4, where the median (nominal present/absent cutoff) for all genes on the chip was set to 500. Where multiple probesets were present (n = 4 for Lck, n = 2 for Cd4), the corresponding probeset values were averaged. Reporter activity is derived from Figure 1, but is expressed as a percentage of maximum (without error bars) for comparison purposes. Only Cd4[Cre] activity appears to closely reflect the activity of its endogenous counterpart, while Cd2[Cre] activity is dramatically accelerated, and Lck[Cre] of either strain is substantially delayed. (PDF) [file pone.0046590.s001.pdf]

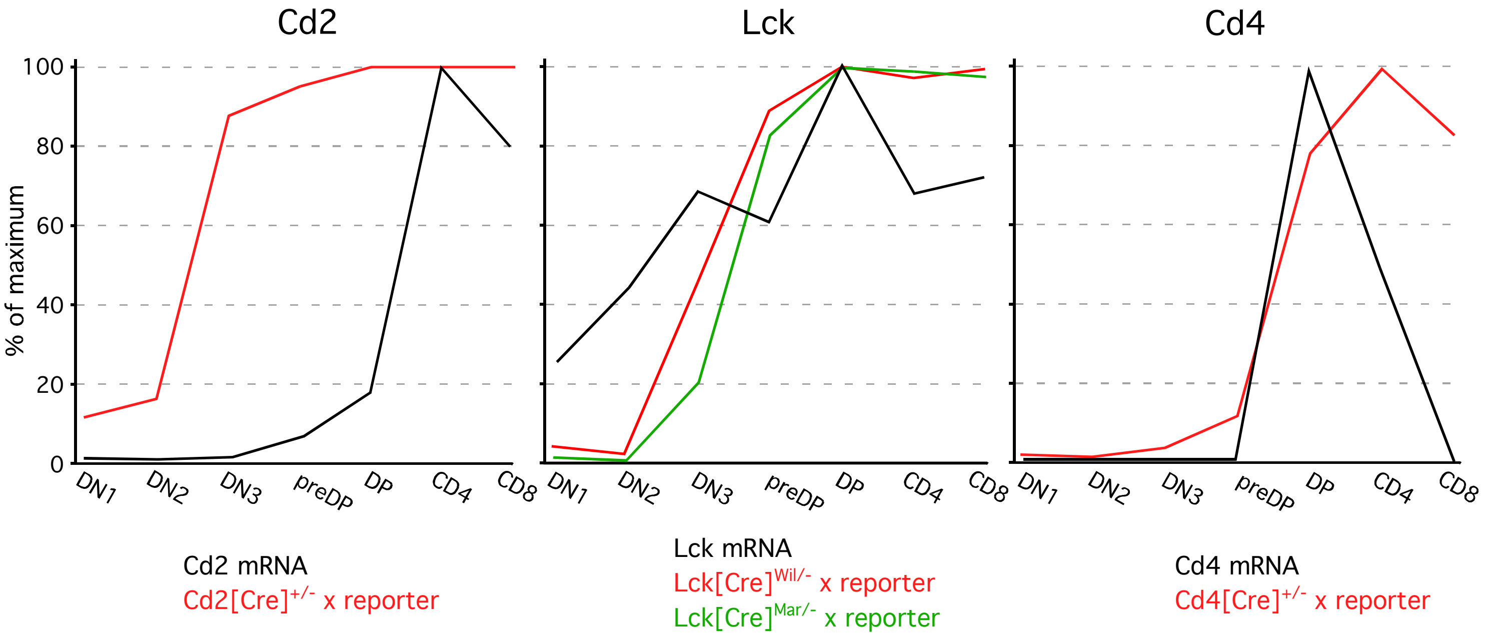

Supplemental Figure 1. Temporal comparison of endogenous gene transcription vs. Cre transgene activity. RNA was isolated from pooled, sorted C57BL/6 thymocyte populations, as shown, and used as template to probe MOE4302 gene arrays (Affymetrix, representing all known genes and ESTs). Gene expression data represent the mean MAS5 value for three independent gene chips at each stage, expressed as a percentage of maximum expression. Absolute maximum values were 18,142 for Cd2, 12,907 for Lck, and 3782 for Cd4, where the median (nominal present/absent cutoff) for all genes on the chip was set to 500. Where multiple probesets were present (n = 4 for Lck, n = 2 for Cd4), the corresponding probeset values were averaged. Reporter activity is derived from Figure 1, but is expressed as a percentage of maximum (without error bars) for comparison purposes. Only Cd4[Cre] activity appears to closely reflect the activity of its endogenous counterpart, while Cd2[Cre] activity is dramatically accelerated, and Lck[Cre] of either strain is substantially delayed.
